# Catalytic Hydrodeoxygenation of Bio-oil Model Compounds over Pt/HY Catalyst

**DOI:** 10.1038/srep28765

**Published:** 2016-06-30

**Authors:** Heejin Lee, Hannah Kim, Mi Jin Yu, Chang Hyun Ko, Jong-Ki Jeon, Jungho Jae, Sung Hoon Park, Sang-Chul Jung, Young-Kwon Park

**Affiliations:** 1 School of Environmental Engineering, University of Seoul, Seoul 02504, Korea; 2 School of Chemical Engineering, Chonnam National University, Gwangju 61186, Korea; 3 Department of Chemical Engineering, Kongju National University, Cheonan 31080, Korea; 4 Clean Energy Research Center, Korea Institute of Science and Technology, Seoul 02792, Korea; 5 Department of Clean Energy and Chemical Engineering, Korea University of Science and Technology, Daejeon 34113, Korea; 6 Department of Environmental Engineering, Sunchon National University, Suncheon 57922, Korea

## Abstract

The hydrodeoxygenation of a model compound of lignin-derived bio-oil, guaiacol, which can be obtained from the pyrolysis of biomass to bio-oil, has attracted considerable research attention because of its huge potential as a substitute for conventional fuels. In this study, platinum-loaded HY zeolites (Pt/HY) with different Si/Al molar ratios were used as catalysts for the hydrodeoxygenation of guaiacol, anisole, veratrole, and phenol to a range of hydrocarbons, such as cyclohexane. The cyclohexane (major product) yield increased with increasing number of acid sites. To produce bio-oil with the maximum level of cyclohexane and alkylated cyclohexanes, which would be suitable as a substitute for conventional transportation fuels, the Si/Al molar ratio should be optimized to balance the Pt particle-induced hydrogenation with acid site-induced methyl group transfer. The fuel properties of real bio-oil derived from the fast pyrolysis of cork oak was improved using the Pt/HY catalyst.

Bio-oil obtained by the pyrolysis of lignocellulosic biomass contains a range of chemicals, such as acids (e.g. acetic acid), anhydrosugars (e.g. levoglucosan), furanics (e.g. furan, furfural), phenolics (e.g. guaiacol), aldehydes, and ketones^1^,^2^,^3^,^4^,^5^,^6^. These bio-based chemicals and bio-based fuels are potential alternatives to petroleum-based chemicals and fuels. In particular, a high bio-oil yield from biomass can be achieved using a fast-pyrolysis technique. Bio-oil has potential use as a fuel, such as gasoline, diesel or jet fuel, but it needs to be up-graded by removing oxygen before it can find widespread use. Among the many oxygen removal techniques, a catalytic process is believed to be most efficient. The catalytic processes for oxygen removal can be classified into two areas, atmospheric catalytic fast pyrolysis and high pressure hydrodeoxygenation (HDO). Catalytic fast pyrolysis uses a series of microporous zeolites (HZSM-5, HY, HBeta, etc.) and mesoporous materials (Al-SBA-15, Al-MCM-48, Al-MCM-41, etc.) in the absence of hydrogen. Although high pressure HDO consumes considerable amounts of hydrogen during the reactions, its ability to remove oxygen from bio-oil by HDO is much better than that by atmospheric catalytic fast pyrolysis. Furthermore, in the HDO process, high pressure hydrogenation can prevent coke deposition on the catalyst surface, which would be beneficial for the reactor operation^7^,^8^. HDO has attracted considerable attention because the product quality is even better than conventional transportation fuel^7^,^8^.

Among the various components of bio-oil, a series of phenolic compounds originating from lignin comprises 20 ~ 30 wt.% of the organic products^9^. In the bio-oil produced from the fast-pyrolysis of lignin, the proportion of guaiacol and its derivatives is approximately 39%. Among the various compounds, guaiacol is considered a representative model compound for bio-oil originating from lignin because it has two types of C-O bonds (Csp_2_OH and Csp_2_OCH_3_) in its molecular structure^8^. Therefore, many studies have used guaiacol as a model compound and examined the HDO of guaiacol using a range of catalysts, such as sulfided NiMo/Al_2_O_3_ and CoMo/Al_2_O_3_, which are well-known catalysts for hydrodesulphurization, precious metal catalysts (platinum, ruthenium, rhodium, etc.), and nickel (Ni) catalyst. Precious metal catalysts exhibit higher catalytic activities than sulfided NiMo/Al_2_O_3_ and CoMo/Al_2_O_3_^10^.

In addition to these catalytically-active metallic components, the catalyst support also plays an important role. In particular, acidic supports have been reported to enhance the catalytic activity for deoxygenation in the HDO reaction^8^,^11^. Therefore, a bifunctional catalyst that combines the hydrogenation ability of a metal with the deoxygenation ability of acid sites, has attracted considerable attention for HDO. Hong *et al*. suggested that a bi-functional catalyst, Pt/HY, is a suitable catalyst for the HDO of phenol^9^. Zhu *et al*. used Pt/HBeta as a catalyst for the HDO of anisole^12^. They reported that platinum and acid sites had a synergistic effect for HDO. Lee *et al*. reported that an acidic support, SiO_2_-Al_2_O_3_, enhanced the production of cyclohexane when Rh/SiO_2_-Al_2_O_3_ was used as a catalyst^11^. Nimmanwudipong *et al*. used Pt/Al_2_O_3_ as a catalyst for the hydroconversion of guaiacol, and reported that this bi-functional catalyst enhances HDO^13^.

Although many studies have reported the advantages of bifunctional catalysts, there is a paucity of reports on the bi-functional catalyst Pt/HY for the HDO of guaiacol. In this study, a series of Pt/HY catalysts with different silicon to aluminum (Si/Al) molar ratios were used for the HDO of guaiacol. The reaction mechanism for the HDO of guaiacol was examined by performing the HDO of the main reaction intermediates of guaiacol HDO, i.e., veratole, phenol and anisole. In addition to the model compound reactions, HDO of the actual bio-oil obtained from the pyrolysis of cork oak was also carried out to evaluate the feasibility of Pt/HY for practical applications.

## Results and Discussion

### Characterization of the catalysts

Table S1 lists the physical properties of the catalysts used in this experiment. In the case of using HY as a support, the surface area and pore volume of the Pt/HY catalysts [Pt/HY(2.6), Pt/HY(40), Pt/HY(100)] increased with increasing Si/Al molar ratio of the HY support. The BET surface areas of Pt/HY(2.6), Pt/HY(40) and Pt/HY(100) were 527, 657, and 697 m^2^/g, respectively, and the corresponding pore volumes were 0.33, 0.47, and 0.50 cm^3^/g. When HZSM-5 was used as a support, the BET surface area (335 m^2^/g) and pore volume (0.29 cm^3^/g) of Pt/HZSM-5 were the lowest among the catalysts used in this study.

Figure S1 presents the NH_3_-TPD spectra of Pt/HY with different Si/Al molar ratios and Pt/HZSM-5. For the series of Pt/HY catalysts with different Si/Al molar ratios, a single major peak appeared. Considering that the peak areas in the TPD spectra are related to the total amount of ammonia desorbed from the acid sites, the total number of acid sites decreased with increasing Si/Al molar ratio. In the case of using HY(2.6) as a support, a distinct peak appeared at 180 °C together with a weak broad peak at approximately 300 °C, indicating the presence of both weak and strong acid sites. This means that an increase in the amount of aluminum incorporated in Y zeolite resulted in an increased number of Brönsted and Lewis acid sites. In contrast to the Pt/HY catalysts, Pt/HZSM-5 showed two distinct peaks, a low-temperature peak around 180 °C and a high-temperature peak at approximately 340 °C, which is higher than that of Pt/HY and was more distinct. This indicates that the acid strength of Pt/HZMS-5 can be stronger that of Pt/HY. The acid amount decreased in the order of Pt/HY(2.6) >Pt/HZSM-5> Pt/HY(40) >Pt/HY(100).

### HDO of guaiacol

**Figure 1:**
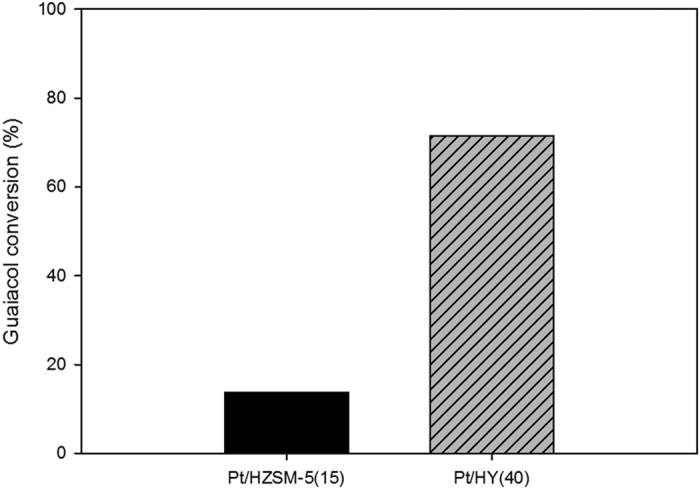


Figure 1 compares the catalytic activities of Pt/HY(40) and Pt/HZSM-5(15) for the HDO of guaiacol. Pt/HY(40) showed higher guaiacol conversion than Pt/HZSM-5(15). In the case of Pt/HZSM-5(15) as a catalyst, guaiacol conversion was as low as 13.8%. Such low conversion might be related to the pore size in their structure because the acid site strength of HZSM-5 was the strongest among the catalysts used.

Guaiacol was unable to penetrate the pores where most of the acid sites were located, because the maximum pore size of HZSM-5 (0.63 nm^14^) was smaller than the kinetic diameter of guaiacol (0.668 nm). Therefore, the small number of acid sites on the external surface of the ZSM-5 particles might participate in the conversion of guaiacol. On the other hand, the diffusion of guaiacol toward the acid sites inside Pt/HY is expected to be easier than that inside Pt/HZSM-5, because HY has a larger pore size (0.74 × 0.74 nm) that is sufficient to allow guaiacol to pass inside the acid sites. The larger pore size of Pt/HY might be the main reason for the better conversion of guaiacol. Hong *et al*. reported that three types of catalysts (Pt/HY, Pt/HZSM-5, and Pt/HBeta) showed high levels of phenol conversion (kinetic diameter: ca. 0.606 nm) in the HDO of phenol^9^. Considering the high conversion of the three catalysts for the HDO of phenol, the difference in the experimental results between previous reports and the present study were attributed mainly to the difference in the molecular size between guaiacol and phenol.

**Figure 2:**
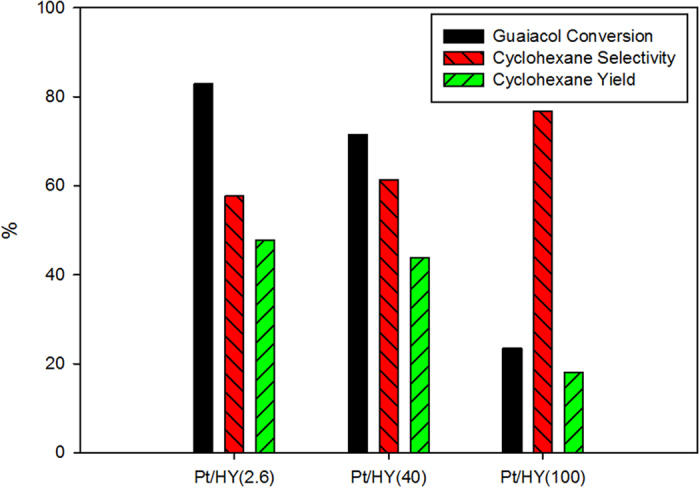


Figure 2 shows the effects of Si/Al on the catalytic activity of 0.5 wt.% Pt/HY. As the Si/Al molar ratio was increased from 2.6 to 100, the level of guaiacol conversion decreased from 82.9% to 23.5%.

The conversion of guaiacol appears to be related to the number of acid sites. The total number of acid sites increased with decreasing Si/Al molar ratio. A larger number of acid sites enhances the possibility for the conversion of guaiacol. Therefore, an increase in guaiacol conversion is due to an increase in the number of acid sites. Zhu *et al*. reported that bifunctional Pt/H-Beta, which contains both strong acid sites and metal particles, has a three-fold higher turn-over frequency for the HDO of anisole than Pt/SiO_2_, which possesses few weak acid sites^12^. They insisted that the Brönsted acid sites near the Pt particles have a synergistic effect in enhancing the HDO of anisole. Lee *et al*.^11^ reported that the acidity of the support affected the catalytic activity of the bifunctional Rh catalyst for the hydroconversion of guaiacol. In this study, a decrease in the Si/Al molar ratio of Pt/HY increased the conversion of guaiacol. The Brönsted acid sites near the Pt particles might be related to the synergistic effect because a decrease in Si/Al molar ratio means an increase in the number of acid sites in Pt/HY. The experimental results are in good agreement with previous results.

Table 1 lists the major compounds produced from the HDO of guaiacol. The most abundant compound was cyclohexane. Considering the high octane number of cyclohexane (~83), the bio-oil produced from the catalytic conversion by Pt/HY might be a promising alternative transportation fuel (gasoline). Ahmad *et al*.^15^ also reported that cyclohexane compounds in bio-oil produced from the HDO of biomass are potential alternative transportation fuels.

In addition to the production of mono-cyclic cyclohexane, bicyclic 1,1′-biyclohexyl was also observed according to the Si/Al molar ratio of Pt/HY. Considering the future use of bio-oil as an alternative or complementary fuel to diesel and jet fuel, the proportion of cyclopentylmethyl-cyclohexane and 1,1′-biyclohexyl (cetane number: 51) in bio-oil might be a crucial factor. In this study, Pt/HY(40) produced more bicyclic 1,1′-biyclohexyl than Pt/HY(2.6). The high density of acid sites [Pt/HY(2.6)] might prefer mono-molecular dehydration for mono-cyclic cyclohexane over bimolecular alkylation for bicyclic 1,1′-bicyclohexyl^16^. The combination of Brönsted acid sites and local confinements in zeolite is a critical factor for the formation of bicyclic compounds. In particular, large pore zeolites (Y and beta) showed higher catalytic activities because of their pore shapes and sizes^16^,^17^. Zhao *et al*. reported that palladium supported on H-beta zeolite exhibited selective catalytic hydroalkylation and deoxygenation to produce bicycloalkanes^16^.

Based on the experimental results and previous reports, the ratio of metal sites to acid sites should be optimized to determine the proper quality of bio-oil from the HDO of biomass.

### HDO of phenol, anisole and veratrole

As listed in Table 1, the HDO of guaiacol at 250 °C produced a range of products, such as veratrole (1,2-dimethoxybenzene), anisole (methoxybenzene), 2-methoxy-4-methylphenol, phenol, methylcyclopentane, and 1,1-bicyclohexyl, in addition to the main product, cyclohexane. Among other product components, veratrole, anisole, cresol, and phenol are well-known reaction intermediates of the catalytic conversion processes of guaiacol^13^,^18^,^19^,^20^. The main reactions of the HDO of guaiacol include direct hydrogenation, dehydration, direct deoxygenation, demethylation, methylation, and transalkylation^19^,^21^. Anisole is produced when the hydroxyl group (−OH) of guaiacol is removed by deoxygenation, whereas phenol is produced from the deoxygenation of catechol, which is formed by the demethylation of guaiacol, or from the direct demethoxylation of guaiacol. The demethylation of anisole also produces phenol^22^. Veratrole, which is an early reaction intermediate of the HDO of guaiacol, is reportedly produced from the intramolecular methyl transfer of guaiacol^23^. Cresol is formed from the dehydration of methylcatechol and the methylation of phenol as well as from the transalkylation of anisole^20^,^24^. In this study, however, catechol was not detected. According to Zhao *et al*.^25^, catechol can be detected only when the reaction duration is short. They reported that catechol was detected when the reaction duration was controlled to 0.3 min using a packed bed reactor. The detection of catechol with a high reaction rate would be difficult in such a batch reactor, as used in this study.

**Figure 3:**
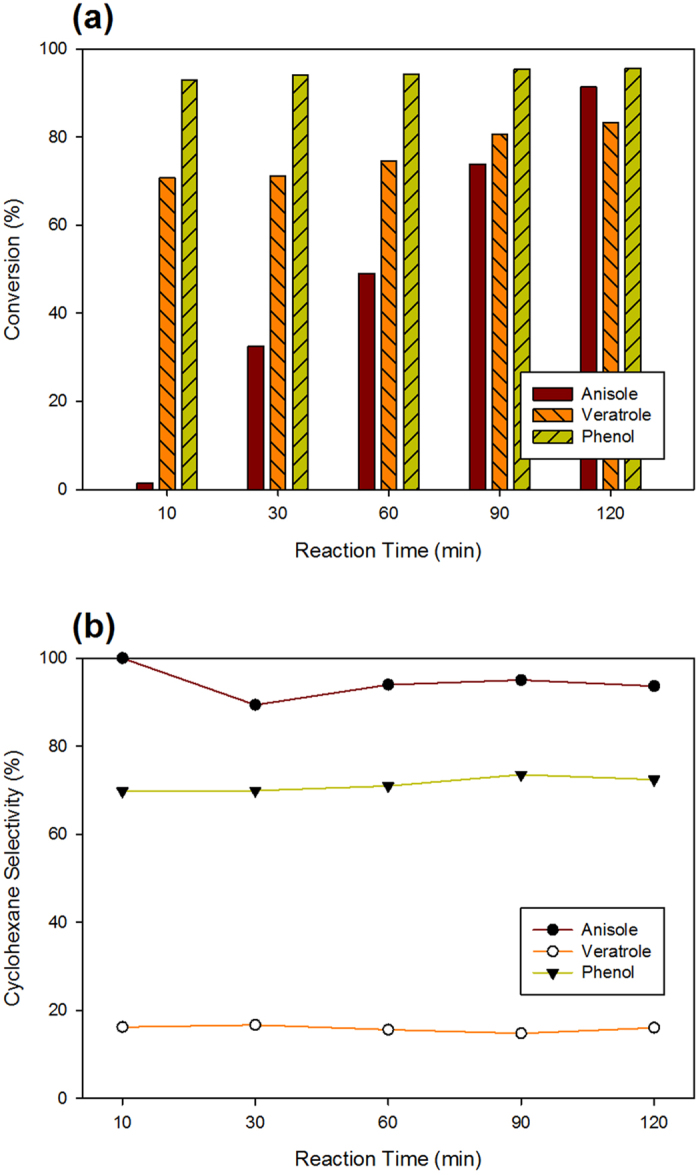
Effect of reaction time on (**a**) the conversion of anisole, veratrole and phenol, (**b**) the selectivity of cyclohexane over Pt/HY(2.6).

To elucidate the reaction pathway from guaiacol to cyclohexane over Pt/HY catalysts, the HDO of the main reaction intermediate components, anisole, veratrole, and phenol, were performed and the intermediate products of those reactions were analyzed. Figure 3 shows the conversions of phenol, anisole, and veratrole as well as the selectivity toward cyclohexane. The conversion of phenol was very high, 92.9% at 10 min. The conversion of veratrole was also quite high (70.7%), whereas that of anisole was very low (1.4%). The selectivity toward the final product, cyclohexane, from anisole was close to 100%, whereas those from phenol and veratrole were 70% and 15%, respectively.

The main products from the HDO of veratrole were cyclohexane, cyclohexanone, methyl cyclopentane, 4-methylveratrole, anisole, phenol, guaiacol, and methylguaiacols (Table 2). Combined with the results shown in Fig. 3, this suggests that veratrole is converted to cyclohexane via several different reaction pathways. One interesting observation was that a considerable amount of guaiacol was observed as a reaction intermediate of the HDO of veratrole, which in turn is a reaction intermediate of the HDO of guaiacol. This suggests that veratrole produced by the transalkylation of guaiacol may be converted back to guaiacol through dimethylation.

As shown in Fig. 3, the HDO reaction of phenol over the Pt/HY catalyst is very fast and the selectivity toward cyclohexane remains high throughout the reaction time, indicating that equilibrium is reached at an early stage of the reaction. Various bicyclic products, such as cyclohexyl benzene, cyclopentylmethyl cyclohexane, and 1,1′-bicyclohexyl, appeared during the HDO of anisole and phenol, resulting from C-C coupling, e.g., due to aldol condensation^9^,^20^,^26^. Apparently, most of the anisole was converted to cyclohexane with only the marginal production of methyl cyclopentane, methyl cyclohexane, 2,4-dimethyl anisole, 1,1′-bicyclohexyl, and cyclohexyl benzene, suggesting that the reaction pathway from anisole to cyclohexane might be relatively simple.

In addition, the HDO reactions of phenol and anisole were carried out at a lower temperature 150 °C to identify the intermediates more reliably. The main products of the HDO of phenol for 2 h at 150 °C were cyclohexane (31.09%), cyclohexanone (1.57%), and 1,1′-bicyclohexyl (3.07%). Cyclohexanone, as a product of the hydrogenation of phenol, has been reported repeatedly^27^.

One important product of the hydrogenation of phenol at 150 °C that was not detected in the HDO at 250 °C was cyclohexanol (45.49%). Cyclohexanol is eventually converted to cyclohexane via consecutive dehydration and hydrogenation^20^. The main products of the HDO of anisole at 150 °C were cyclohexane (63.87%) and 1-methoxycyclohexane (1.41%), the latter of which was not detected in the HDO at 250 °C. 1-Methoxycyclohexane is formed when the benzene ring of anisole is converted to a saturated ring through hydrogenation, as observed previously in low-temperature HDO over Ni/C, Ni/CeO_2_, and Ni/SBA-15^7^. Methoxycyclohexane is converted to cyclohexane through demethoxylation and demethylation. During the HDO of a bio-oil model compound with two functional groups, such as guaiacol and veratrole, C-O cleavage through demethoxylation and dehydration takes place first removing one functional group^28^, which is then followed by saturation of the benzene ring through hydrogenation of the single-functional-group species, such as phenol and anisole, and ensuing demethylation-dehydration that removes the remaining C-O bond.

**Figure 4:**
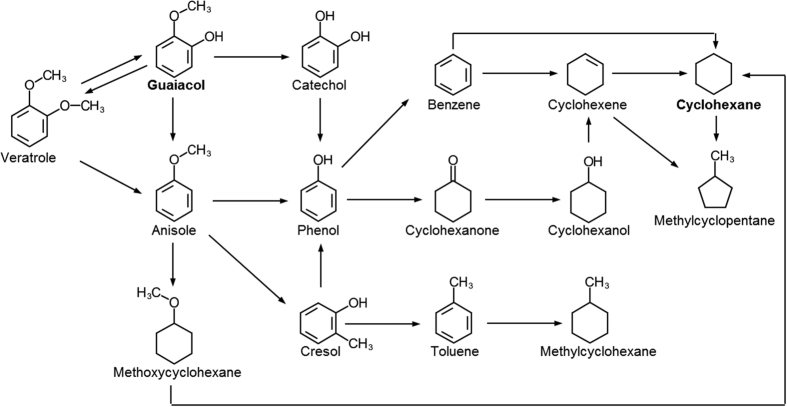


Therefore, the number of functional groups is believed to affect the reaction pathway of the HDO. Figure 4 presents a proposed mechanism for the conversion of guaiacol to cyclohexane over the Pt/HY catalyst deduced from the results of this study and previous reports.

### HDO of Bio-oil

HDO has attracted considerable attention as an alternative to pyrolysis because of the number of drawbacks of bio-oil, including low heating value and carbon content, high oxygen and moisture content, and high viscosity^29^,^30^,^31^. On the other hand, although the catalytic HDO of the lignin monomer, guaiacol, has been studied intensively, there has been little research on the catalytic HDO of actual bio-oil.

Table 3 lists the water content, pH, and viscosity of raw bio-oil and hydrodeoxygenated oil. The cork oak-derived bio-oil used in this study had a typically low pH ranging from 2 to 3, which is similar to those of bio-oils derived from other biomass, such as rice husk^32^, pine sawdust^33^, and miscanthus sinensis^34^. The moisture content was 22.4% and the viscosity was quite high (18.3 cSt). The HDO reaction increased the pH to 4.6 and reduced the viscosity to 2.4 cSt, whereas the moisture content was decreased only slightly.

Elemental analysis of oil is essential to evaluate the oxygen removal of HDO. Table 4 presents the results of elemental analysis and heating value measurements. To reduce the effects of the solvent, light compounds, such as ethanol and ethyl acetate, were removed through vacuum distillation at 70 °C before elemental analysis.

The HDO over 5 wt.% Pt/HY(2.6) reduced the oxygen content from 49.3% to 28.2%, while increasing the carbon content from 41.5% to 61.1%, resulting in a high heating value calculated to be 24.4 MJ/kg. This high oxygen removal efficiency confirms that HDO can be applied to actual bio-oil.

## Conclusions

Platinum-loaded HY zeolites with different Si/Al molar ratios were used as catalysts for the HDO of guaiacol to various hydrocarbons. The yield of cyclohexane, which is the major product of the HDO of guaiacol, increased with decreasing Si/Al molar ratio. The results suggest that the proportion of platinum metal particles to Brönsted acid sites in zeolite needs to be optimized to achieve the maximum production of cyclohexane and alkylated cyclic compounds with a higher octane number. The reaction pathway from guaiacol to cyclohexane was deduced from the results of the HDO of the reaction intermediates (anisole, veratrole, and phenol). HDO of the bio-oil produced from actual cork oak reduced the oxygen content efficiently, leading to an increased heating value.

## Experimental

### Preparation of Pt/HY catalysts

Proton-exchanged Y zeolites with different Si/Al molar ratios (2.6, 40, 100) were used as the catalyst supports. HY(Si/Al = 2.6) and HY(Si/Al = 40) were purchased from Zeolyst, and HY(Si/Al = 100) was obtained from Tosoh Corporation. Platinum was incorporated on these supports by the incipient wetness method using tetraammineplatinum(II) nitrate (Sigma-Aldrich, ≥50.0% Pt basis) as a precursor. After impregnating the platinum precursor, the samples were dried completely at 110 °C for 24 h, and then calcined in air at 500 °C for 3 h to prepare 0.5 wt.% or 5.0 wt.% Pt/HY. HZSM-5(Si/Al = 15) was also purchased from Zeolyst and 0.5 wt.% Pt/HZSM-5 was prepared by the impregnation of tetraammineplatinum(II) nitrate on HZSM-5.

### Characterization of the catalysts

The N_2_ adsorption-desorption isotherms at −196 °C were obtained using an automated gas sorption system (TriStar, Micromeritics). The surface areas of the samples were calculated using the BET (Brunauer-Emmett-Teller) method with a nitrogen partial pressure ranging from 0.05 to 0.2^35^,^36^. The surface acid site properties were determined by the temperature-programmed desorption of NH_3_ (NH_3_-TPD) with a BELCAT (BEL Japan Inc.) equipped with a thermal conductivity detector (TCD) to quantify the ammonia desorbed from the catalyst surface.

### Product analysis

Karl Fischer titration (870 KF Titrino plus, Metrohm) was used to measure the moisture content of the oil. Viscosity measurement was taken using a capillary type viscometer (Cannon) at 40 °C based on ASTM D 445. The pH of bio-oil was measured using a digital pH meter (Starter 300, Ohaus). The C, H and N contents were measured by elemental analysis using the Flash EA 2000 series (Thermo Fisher).

### Reaction conditions for HDO

The catalyst was reduced with hydrogen gas at 500 °C for 3h. For the HDO of guaiacol, 0.4 g of reduced catalyst and 40 ml of a 7.5 wt.% guaiacol (TCI, ≥98.0%) solution in decane (Sigma-Aldrich, ≥99%) were placed in a batch type reactor.

Most (96.8%) of the decane used as the solvent remained unchanged, only producing 0.18% of methyl nonane and 0.05% of dodecane, indicating that it was not involved in the HDO reaction. The inside of the batch type reactor was purged 3 times with high purity hydrogen gas. The total hydrogen pressure of the reactor was then increased to 40 bar. After adjusting the pressure, the reactor temperature was increased from room temperature to 250 °C at 5 °C/min and maintained at 250 °C for 2 h with a stirring speed of 400 rpm.

The HDO experiments of anisole (Sigma-Aldrich, 99.7%), 1,2-dimethoxybenzene (veratole) (Sigma-Aldrich, 99.0%), and phenol (Samchum, 99.0%) were carried out in the same manner as that of guaiacol using 0.5 wt.% Pt/HY(2.6). In addition, the HDO of a bio-oil derived from the fast pyrolysis of actual cork oak was also performed using 5 wt.% Pt/HY (2.6).

Because decane, which is used as the solvent in the HDO of the model compounds, does not dissolve lignin-derived oil, anhydrous ethyl alcohol (Samchun, 99.9%), which is used frequently for the HDO of bio-oil^32^,^33^,^34^ was used instead. Ethanol is not only a solvent but is also involved in the reaction^37^. The mass ratio of cork oak bio-oil and ethanol was 1:1 and the total mass was 40 g. 1.0-g HY(Si/Al = 2.6) with 5 wt.% Pt impregnated was reduced using the same method. While HDO of the model compound used a relatively small mass (7.5 wt.%) of the compound to reveal the reaction pathway accurately, HDO of the actual bio-oil used a larger quantity of oil because bio-oil is composed of a variety of components and fuel quality evaluations through the determination of acidity, viscosity, moisture content, and elemental composition need to be performed. Accordingly, the catalyst mass and its noble metal content were also increased. The reaction duration, temperature and stirring speed were the same as those used for the HDO of model compound. The hydrogen pressure was 70 bar.

After the reaction, the reactor was cooled to room temperature to obtain a liquid sample of the reaction product. These samples were analyzed by gas chromatography/mass spectroscopy (GC/MS, Agilent Technologies). Ultra ALLOY-5 (MS/HT) (5% diphenyl and 95% dimethylpolysiloxane, length 30 m, i.d. 0.25 mm, film thickness 0.5 μm, Frontier Laboratories Ltd. Japan) was used as the metal capillary column.

## Additional Information

**How to cite this article**: Lee, H. *et al*. Catalytic Hydrodeoxygenation of Bio-oil Model Compounds over Pt/HY Catalyst. *Sci. Rep.* **6**, 28765; doi: 10.1038/srep28765 (2016).

## Supplementary Material

Supplementary Information

## Figures and Tables

**Table 1:** Yields (wt%) of main products from HDO of guaiacol over Pt/HY with different Si/Al.

	**Pt/HY(2.6)**	**Pt/HY(40)**	**Pt/HY(100)**
Cyclopentane, methyl	1.94	1.02	
Cyclohexane	47.84	43.87	18.02
Cyclohexane, methyl	3.08		
Cyclohexane, (cyclopentylmethyl)	0.84	1.46	
1-Methoxycyclohexane			1.23
Cyclopentanemethanol			0.75
Benzene, methoxy	1.54	1.29	
Benzene, 1,2-dimethoxy	17.08	28.50	
Phenol	3.18	0.60	
Phenol, 2-methoxy-5-methyl	1.40		
Phenol, 2-methoxy-4-methyl	1.37		
1,1′-Bicyclohexyl	2.07	6.31	0.71

**Table 2:** Yields (wt%) of main products from HDO of anisole, veratrole and phenol over Pt/HY(2.6).

	**Anisole**	**Veratrole**	**phenol**
Cyclopentane, methyl	0.71	0.72	0.35
Cyclohexane	85.57	13.35	69.26
Cyclohexane, methyl	1.37	0.81	
Cyclohexanone			0.21
Cyclohexane, (cyclopentylmethyl)			4.29
Benzene, methoxy		1.38	
Benzene, cyclohexyl	0.34		2.55
Phenol, 2-methoxy		32.19	
Phenol, 2-methoxy-3-methyl		4.97	
Phenol, 2-methoxy-5-methyl		3.74	
Phenol, 2-methoxy-4-methyl		4.66	
3,4-Dimethoxytoluene		4.10	
1,1′-Bicyclohexyl	0.68		12.30

**Table 3:** Physical properties raw bio-oil and HDO bio-oil.

	**raw bio-oil**	**HDO oil**
Water content (%)	22.4	20.1
pH	2.5	4.6
Viscosity (cSt)	18.3	2.4

**Table 4:** Elemental composition and heating value of raw bio-oil and HDO bio-oil.

**Sample**	**Elemental compositions (wt%)**				**H/C**	**O/C**	**HHV**^b^ **(MJ/kg)**
	**C**	**H**	**O**^a^	**N**			
raw bio-oil	41.5	8.1	49.3	1.1	2.3	0.9	18.9
HDO oil	60.1	8.6	28.2	1.5	1.7	0.4	24.4

^a^Calculated by difference.

^b^Calculated on the basis of elemental composition, HHV(MJ/kg) = −1.3675 + 0.3137 C + 0.7009 H + 0.0318 O^38^.
