# Supplementary material for: Catalytic Hydrodeoxygenation of Bio-oil Model Compounds over Pt/HY Catalyst
Source: Sci Rep. 2016 Jun 30;6:28765. doi: 10.1038/srep28765 (PMC4928091; doi:10.1038/srep28765)
Supplement: Supplementary Information [file srep28765-s1.pdf]

Supplementary Information

Catalytic Hydrodeoxygenation of Bio-oil Model Compounds over Pt/HY Catalyst

Heejin Lee[1,a], Hannah Kim[1,a], Mi Jin Yu[1], Chang Hyun Ko[2], Jong-Ki Jeon[3], Jungho Jae[4,5], Sung Hoon Park[6], Sang-Chul Jung[6] & Young-Kwon Park[1,*]

[1] School of Environmental Engineering, University of Seoul, Seoul 02504, Korea

[2] School of Chemical Engineering, Chonnam National University, Gwangju 61186, Korea

[3] Department of Chemical Engineering, Kongju National University, Cheonan 31080, Korea

[4] Clean Energy Research Center, Korea Institute of Science and Technology, Seoul 02792, Korea

[5] Department of Clean Energy and Chemical Engineering, Korea University of Science and Technology, Daejeon 34113, Korea

[6] Department of Environmental Engineering, Sunchon National University, Suncheon 57922, Korea

[a] Co-first authors

*Corresponding author: catalica@uos.ac.kr; parkyk@uos.ac.kr

Table S1. Physical properties of catalysts

	S_{BET} (m^2/g)	V_{total} (cm^3/g)	Si/Al
Pt/HY(2.6)	527	0.33	2.6
Pt/HY(40)	657	0.47	40
Pt/HY(100)	697	0.50	100
Pt/HZSM-5	335	0.29	15

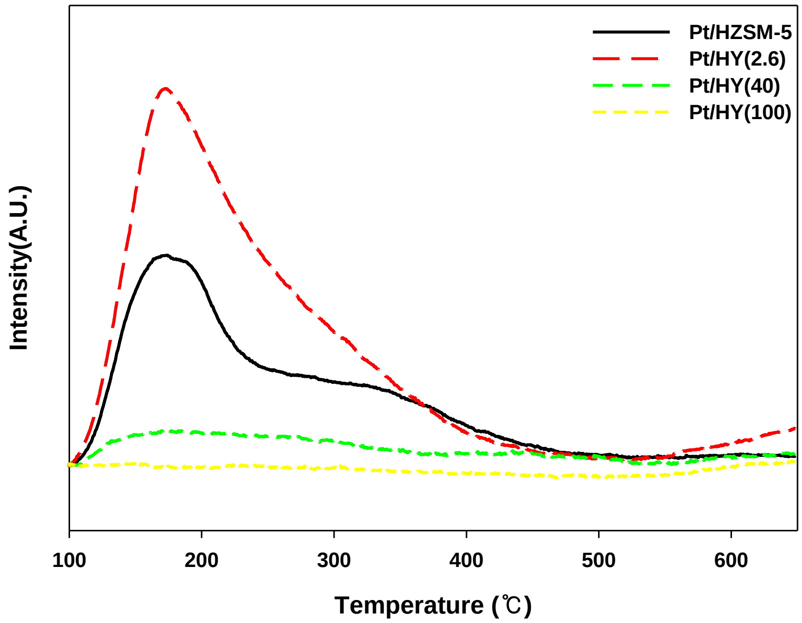

Fig. S1. NH_3 TPD of Pt loaded catalysts
